# Supplementary material for: A systems-based approach to uterine fibroids identifies differential splicing associated with abnormal uterine bleeding
Source: Commun Med (Lond). 2025 Jul 31;5:318. doi: 10.1038/s43856-025-01051-x (PMC12311048; doi:10.1038/s43856-025-01051-x)
Supplement: Supplementary file 2 — Supplementary information [file 43856_2025_1051_MOESM2_ESM.pdf]

# A systems-based approach to uterine fibroids identifies differential splicing associated with abnormal uterine bleeding

Chen-Yi Wang[1]*, Martin Philpott[1]*, Darragh P O'Brien[2], Anne Ndungu[3], Jessica Malzahn[1], Marina Maritati[1], Neelam Mehta[1], Vicki Gamble[1], Beatriz Martinez-Burgo[3], Sarah Bonham[2], Roman Fischer[2], Kurtis Garbutt[3], Christian M Becker[3], Sanjiv Manek[3], Adrian L Harris[4], Frank Sacher[5], Maik Obendorf[5], Nicole Schmidt[5], Jörg Müller[5], Thomas M. Zollner[5], Krina T Zondervan[3], Benedikt M Kessler[2], Udo Oppermann[1], Adam P Cribbs[1]

## LIST OF SUPPEMENTARY MATERIALS

**Supplementary Fig. 1** Overview of tissue collection in the study.

**Supplementary Fig. 2** Identification of fibroid-associated gene mutations using SureSelect targeted sequencing.

**Supplementary Fig. 3** Features and enriched pathways that highlight potential impacts of fibroid presence on endometrium.

**Supplementary Fig. 4**. Multi-omics analysis of myometrium and UFs.

**Supplementary Fig. 5** Proteomic data comparison of fibroid and myometrium tissues in patients with UFs.

**Supplementary Fig. 6** Multi-omics analysis of UFs.

**Supplementary Fig. 7** Sequence alignment, domain composition and predicted structures of TGFBR2 protein isoforms.

**Supplementary Fig. 8** Batch correction of the integrated data of healthy and UF endometrium.

**Supplementary Fig. 9** Quality check of the integrated data of healthy and UF endometrium.

**Supplementary Fig. 10** Differences of cell type composition between healthy and UF endometrium datasets at single cell level.

**Supplementary Fig. 11** Collagen and laminin signalling in healthy and UF endometrium at single cell level.

**Supplementary Fig. 12** Comparison of signalling pathways in healthy and UF tissues.

**Supplementary Fig. 13** Enriched pathways of genes involved in THESC decidualization process identified by using bulk short read and long read sequencing

**Supplementary Fig. 14** Enriched pathways of genes with alternative isoform usage identified in TGF-β treated THESC during decidualisation.

**Supplementary Fig. 1: Overview of Tissue Collection in the Study.**

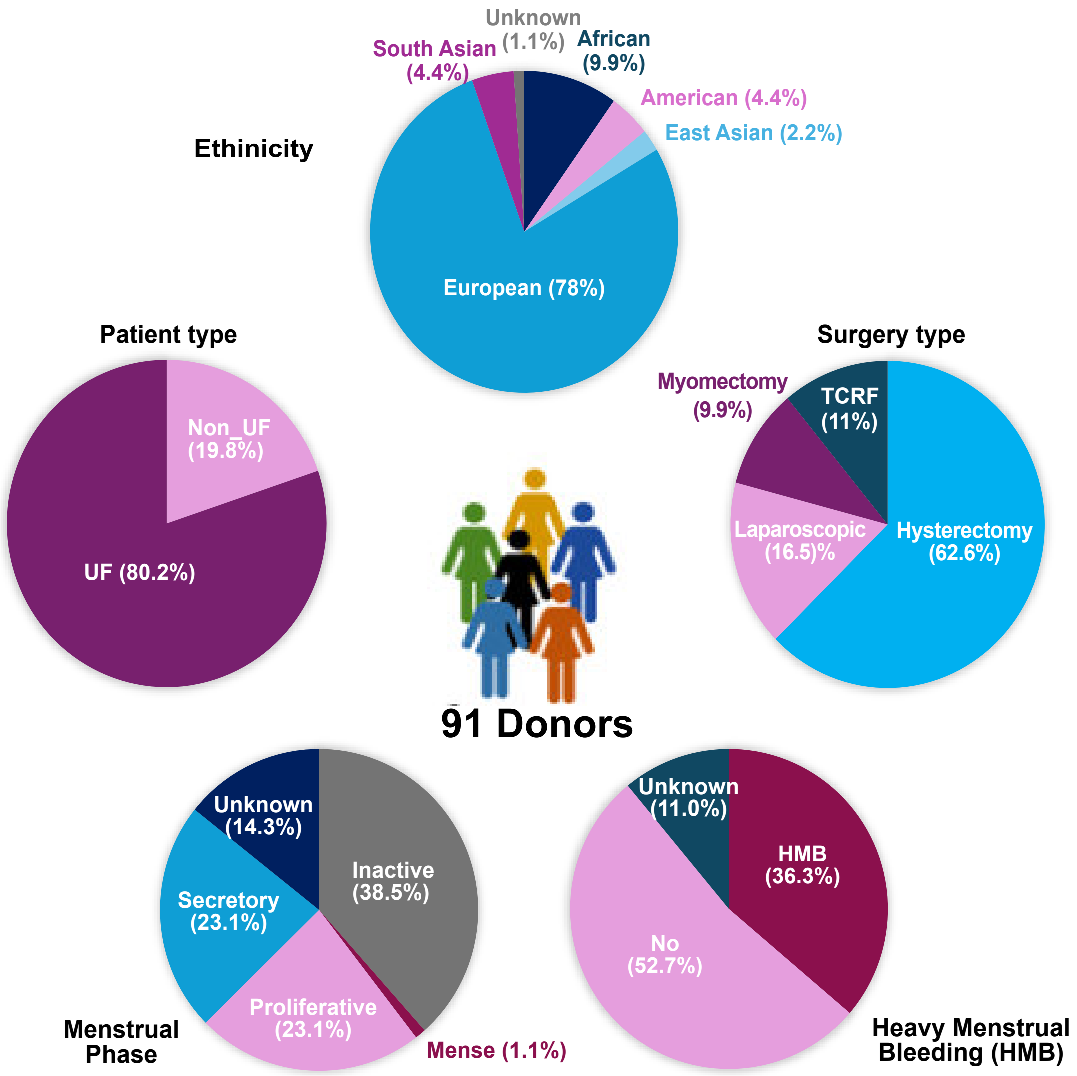


**Supplementary Fig. 1 Overview of Tissue Collection in the Study.** This schematic diagram illustrates the classification of the patient cohort based on the type of surgery undertaken, the distinction between patients with fibroids and control subjects without fibroids, the status of heavy menstrual bleeding (HMB), and the phase of the menstrual cycle.

## Supplementary Fig. 2: Identification of fibroid-associated gene mutations using SureSelect targeted sequencing.

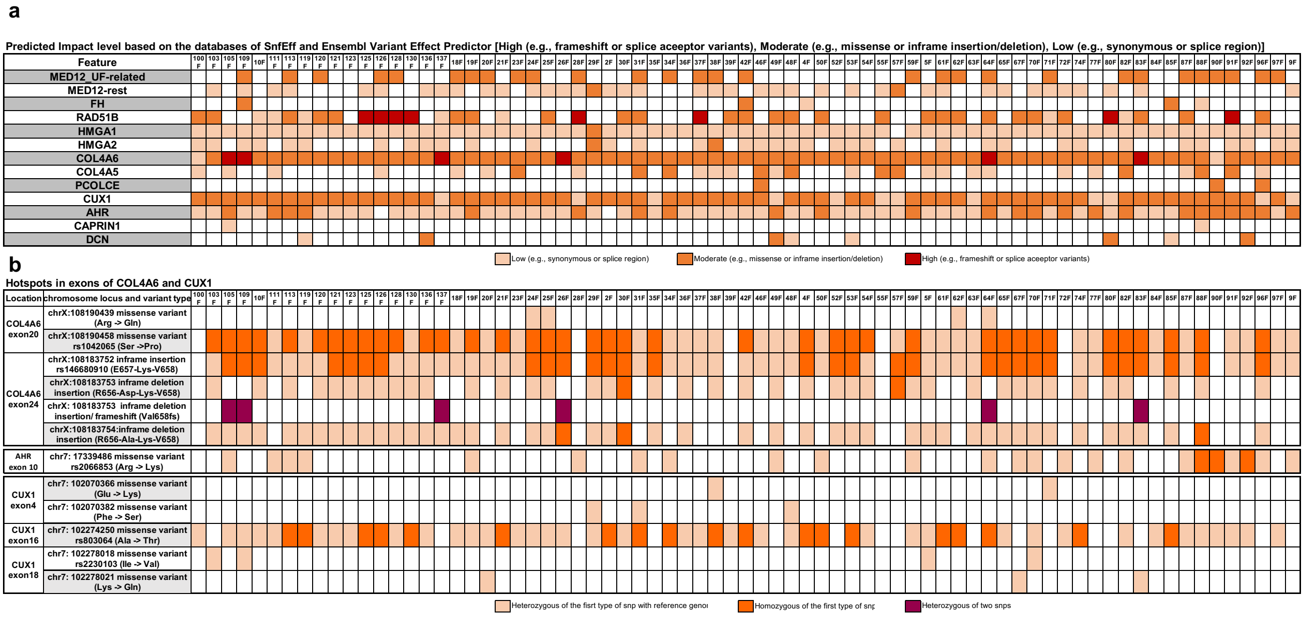


**Supplementary Fig. 2 Identification of fibroid-associated gene mutations using SureSelect targeted sequencing.**

**a** Detected variants within fibroid-associated genes across sampled tissues, identified via SureSelect targeted sequencing. The predicted impact of each variant on protein-coding, as identified by SnpEff and the Ensembl Variant Effect Predictor, is classified as high (red), moderate (orange) or low (light orange). **b** Genomic locus of mutation hotspots identified in *COL4A6*, *AHR* and *CUX1*.

**Supplementary Fig. 3: Features and enriched pathways that highlight potential impacts of fibroid presence on endometrium.**

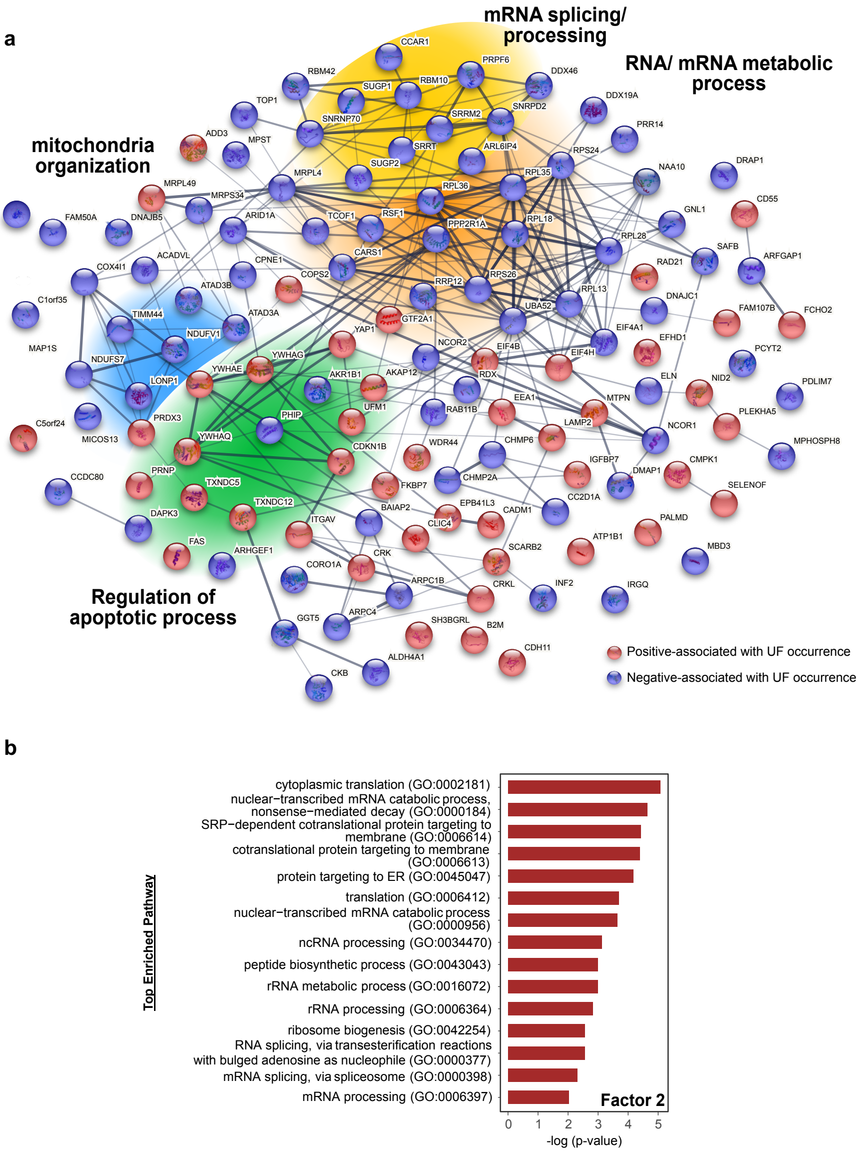


**Supplementary Fig. 3 Features and enriched pathways that highlight potential impacts of fibroid presence on endometrium. a** STRING diagrams elucidate features associated with Factor 2, providing insight into the molecular mechanisms underpinning the observed patterns. **b** Gene ontology pathway analysis highlights pathways that are associated with fibroid presence.

**Supplementary Fig. 4: Multi-omics analysis of myometrium and UFs.**

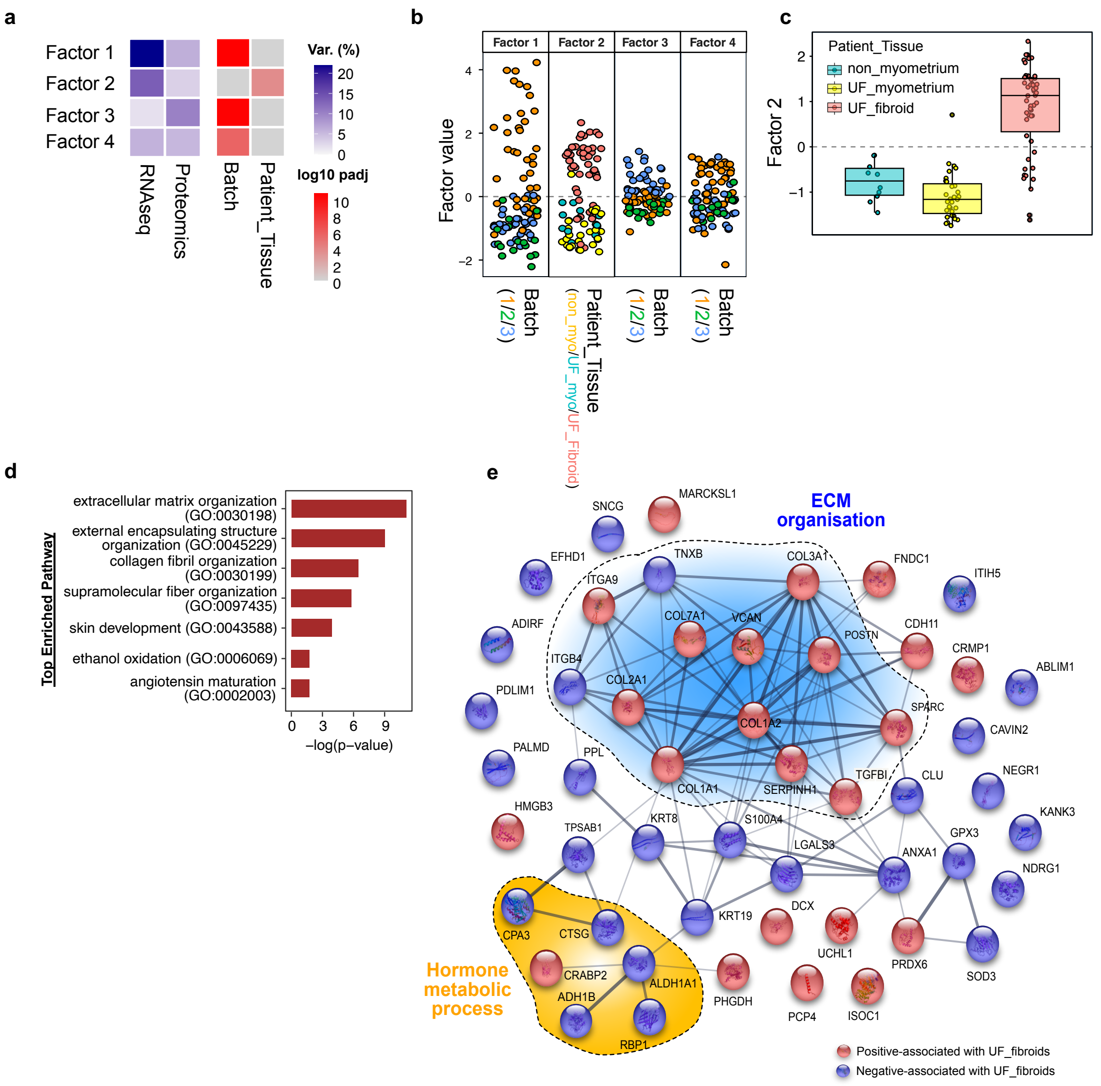


**Supplementary Fig. 4 Multi-omics analysis of myometrium and UFs**

**a** Left: The relative contribution of the transcriptomic and proteomic datasets to MOFA factors, expressed as a percentage of variance. Right: Correlation of variance with specified parameters ($Log_{10}$padj). **b** Scatter plot illustrating the differentiation of sample groups in the indicated latent factor. The MOFA factor value represents the relative positioning of samples, where a higher absolute factor value suggesting a stronger effect. **c** Boxplots showing the distribution of sample groups in Factor 2 that correlates with tissue type. The centre line represents the median, the lower and upper hinges correspond to the 25$^{th}$ and 75$^{th}$ percentiles, and whiskers extend to 1.5 times the interquartile range. **d** Gene ontology pathway analysis highlighting enriched pathways identified in Factor 2. **e** STRING network diagrams of features associated with Factor 2.

**Supplementary Fig. 5: Proteomic data comparison of fibroid and myometrium tissues in patients with UFs.**

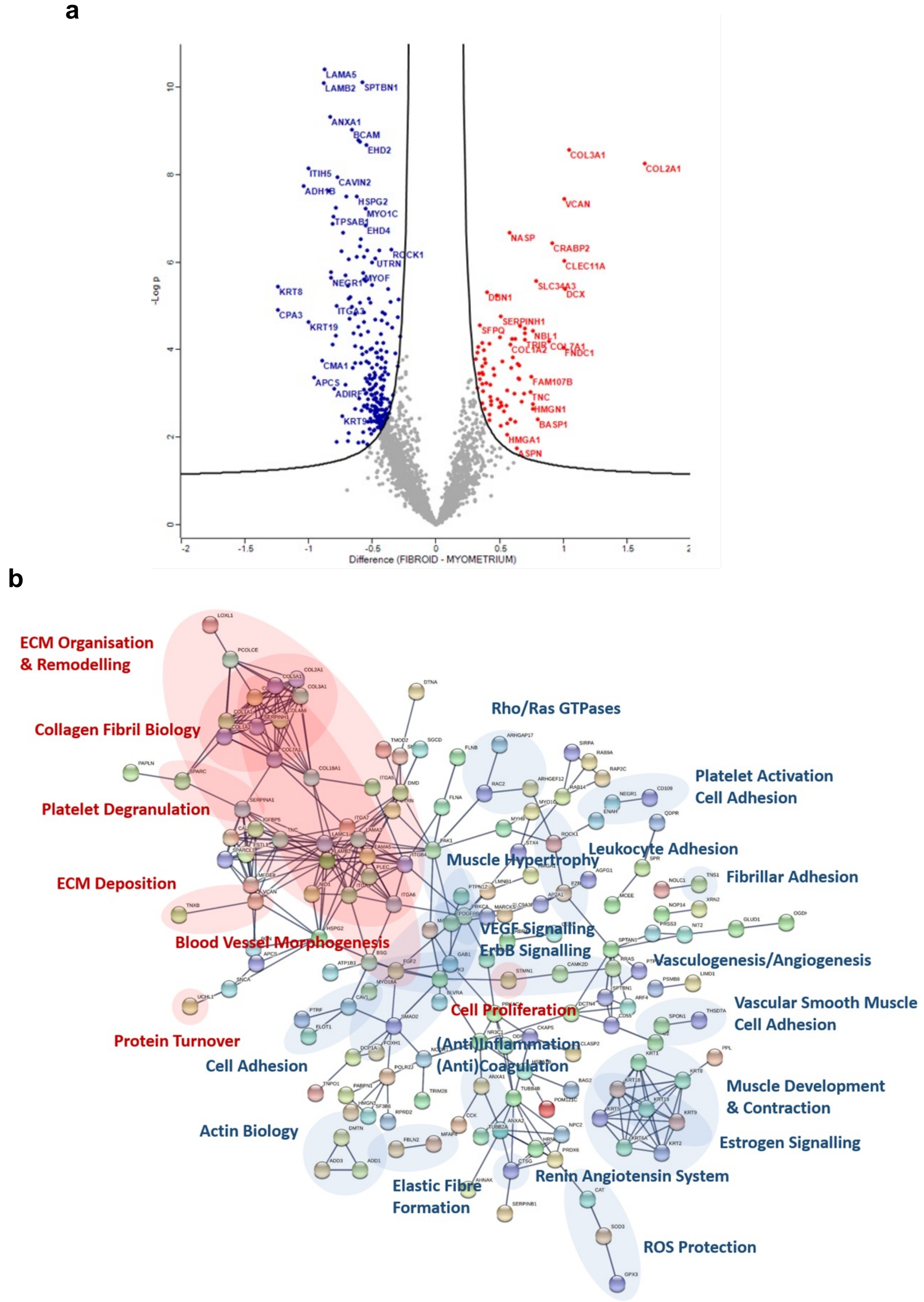


**Supplementary Fig. 5 Proteomic data comparison of fibroid and myometrium tissues in patients with UFs. a** Volcano plot of UF versus myometrium tissues at 1% FDR. Up-regulated proteins in UF compared to myometrial controls are coloured red, while their down-regulated counterparts are coloured blue. **b** STRING differential pathway analysis.

## Supplementary Fig. 6: Multi-omics analysis of UFs

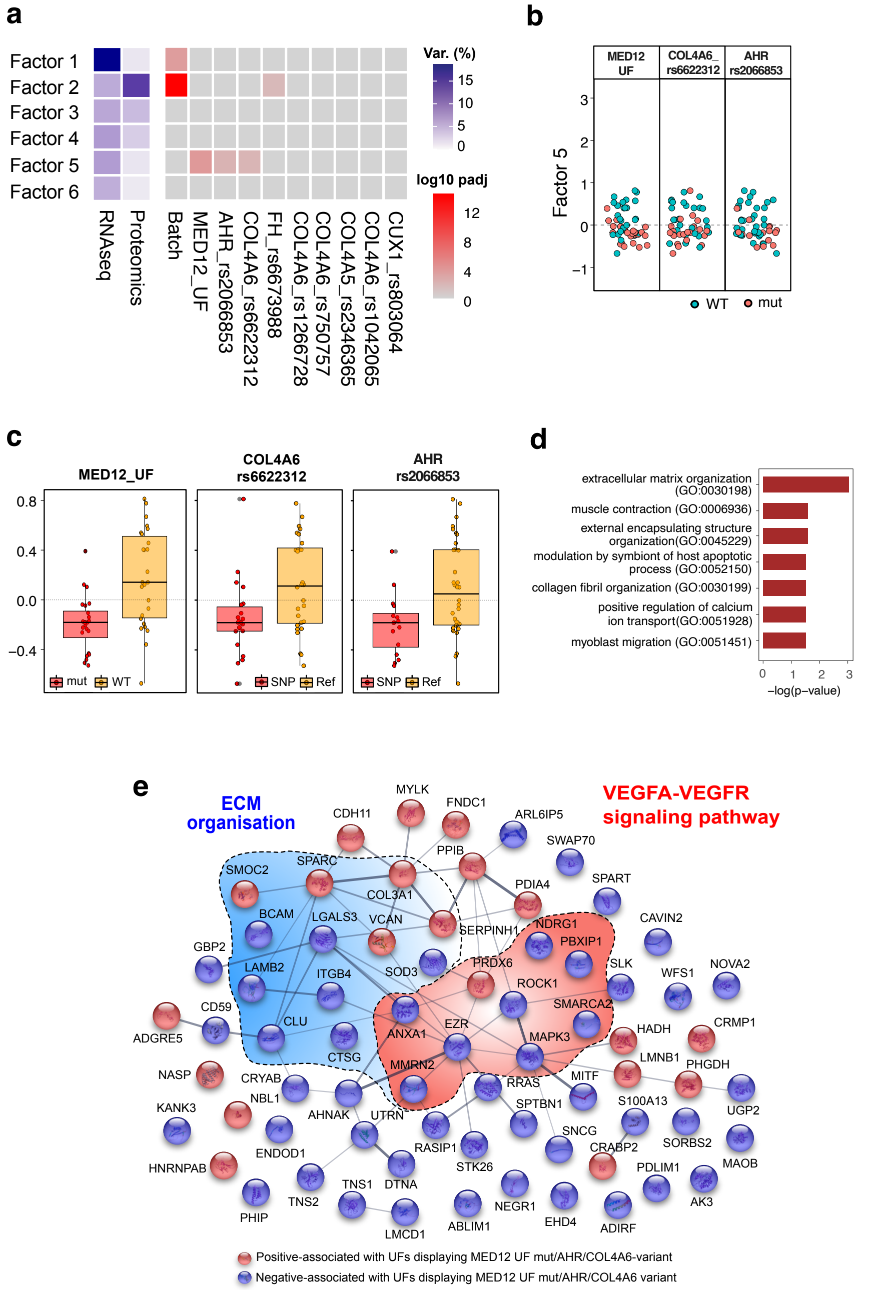


**Supplementary Fig. 6 Multi-omics analysis of UFs**

**a** Left: The relative contribution of the transcriptomic and proteomic datasets to MOFA factors, expressed as a percentage of variance. Right: Correlation of variance with specified parameters ($Log_{10}$padj). **b** Scatter plot illustrating the differentiation of sample groups based on key clinical and genetic parameters, including *MED12* UF mutations (wt, n=26; mut, n=24), *COL4A6* rs6622312 (wt, n=28; mut, n=22), and *AHR* rs2066853 (wt, n=34; mut, n=16) . MOFA factor values represent the relative positioning of samples, where a higher absolute factor value suggesting a stronger effect. **c** Boxplots showing the distribution of sample groups in Factor 5 that correlates with driver mutations. The centre line represents the median, the lower and upper hinges correspond to the $25^{th}$ and $75^{th}$ percentiles, and whiskers extend to 1.5 times the interquartile range. **d** Gene ontology pathway analysis highlighting enriched pathways identified in Factor 5. **e** STRING network diagrams of features associated with Factor 5.

## Supplementary Fig. 7: Sequence alignment, domain composition and predicted structures of TGFBR2 protein isoforms

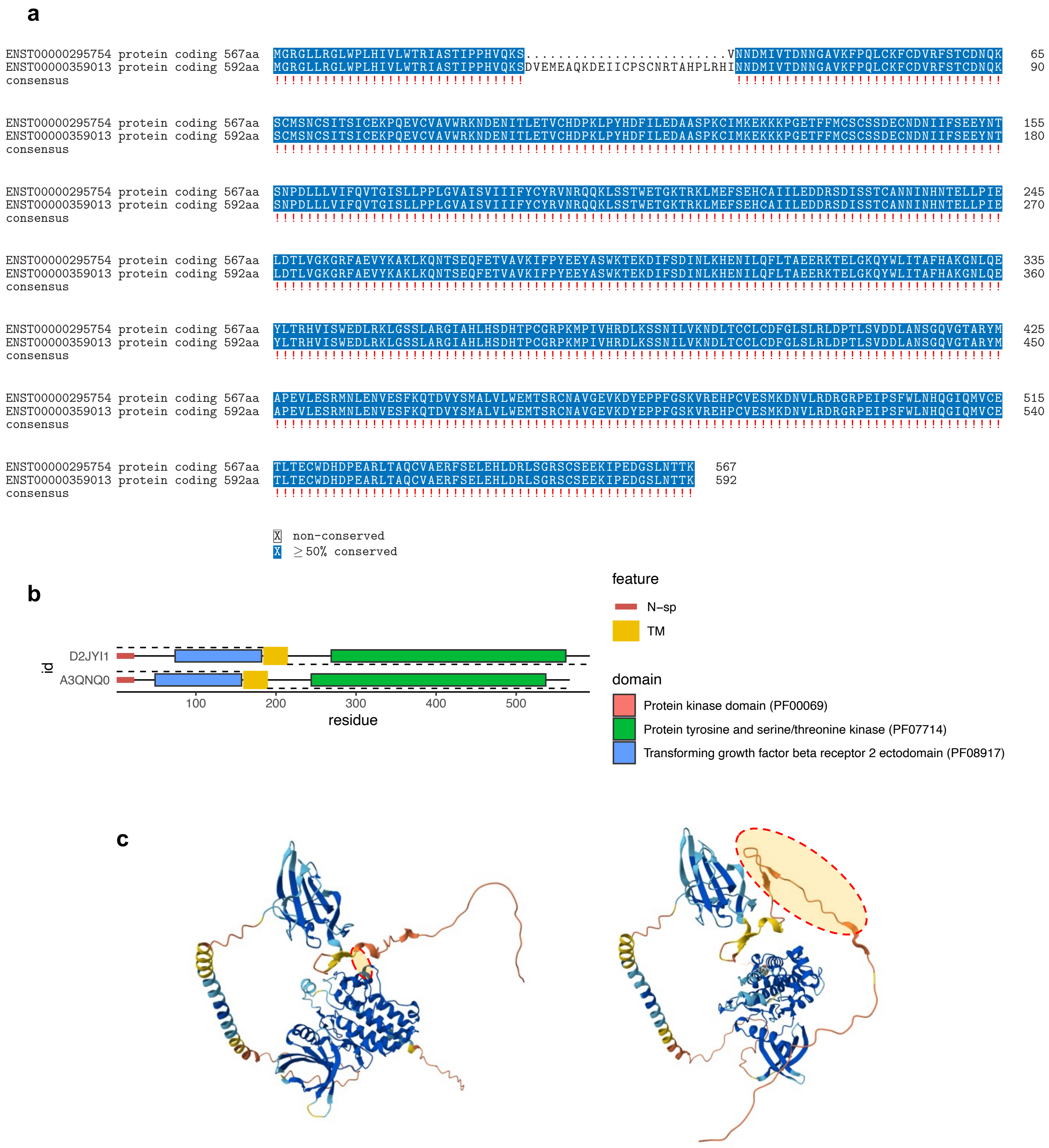


**Supplementary Fig. 7 Sequence alignment, domain composition and predicted structures of TGFBR2 protein isoforms. a** Protein sequence alignment. **b** conserved domain search and **c** predicted protein structures using AlphaFold 3.

**Supplementary Fig. 8: Batch correction of the integrated data of healthy and UF endometrium.**

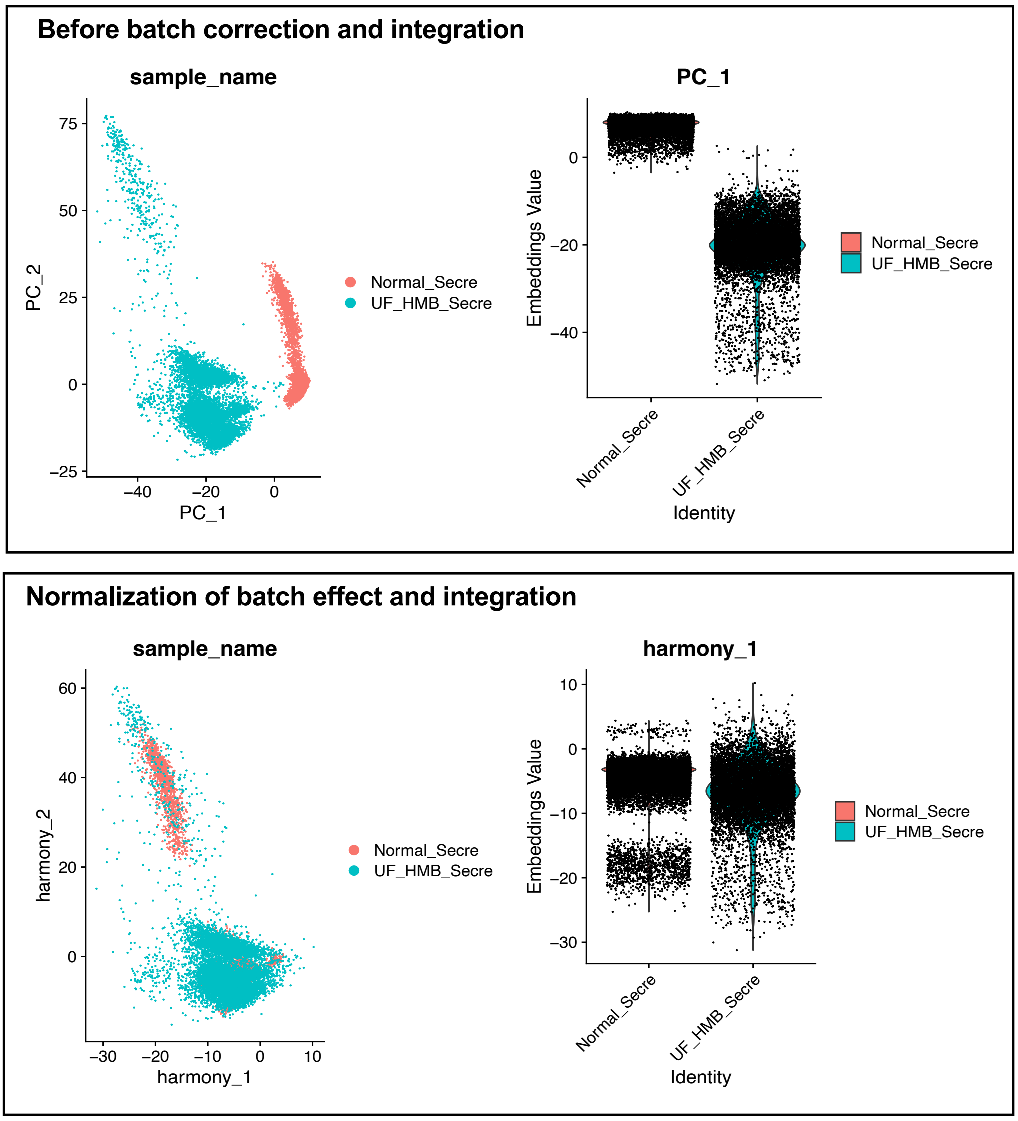


**Supplementary Fig. 8 Batch correction of the integrated data of healthy and UF endometrium.**
PCA plot in PC1-PC2 and the harmony embedding shows the batch effect normalization, before (upper panel) and after (bottom panel) the integration.

**Supplementary Fig. 9: Quality check of the integrated data of healthy and UF endometrium.**

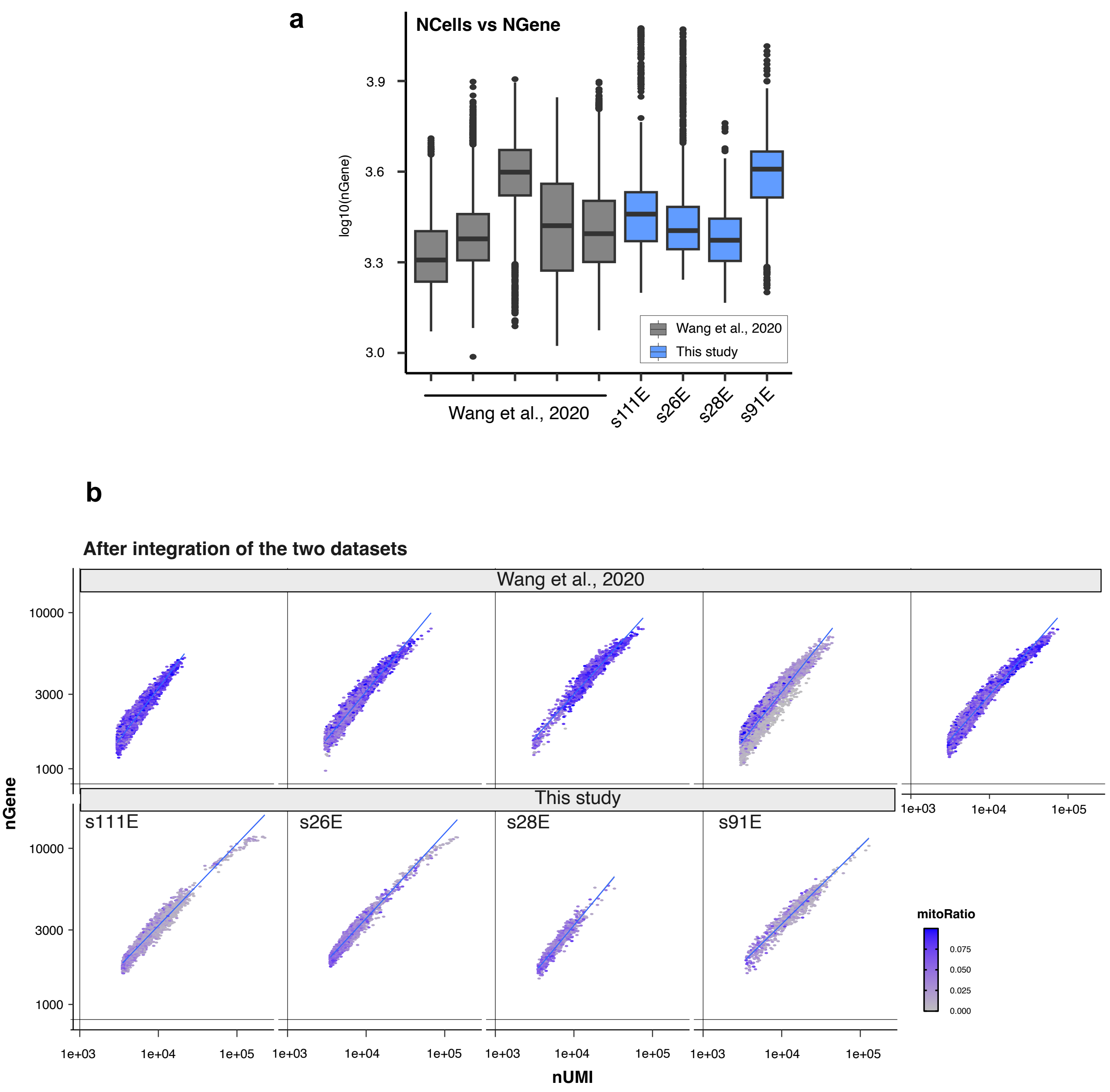


**Supplementary Fig. 9 Quality check of the integrated data of healthy and UF endometrium**
**a** Boxplot of the gene distribution per cell in each sample. **b** Scatter plot of number of genes detected and number of UMIs per cell in each sample. Fraction of mitochondria reads was coloured in purple.

**Supplementary Fig. 10: Differences of cell type composition between healthy and UF endometrium datasets at single cell level.**

a

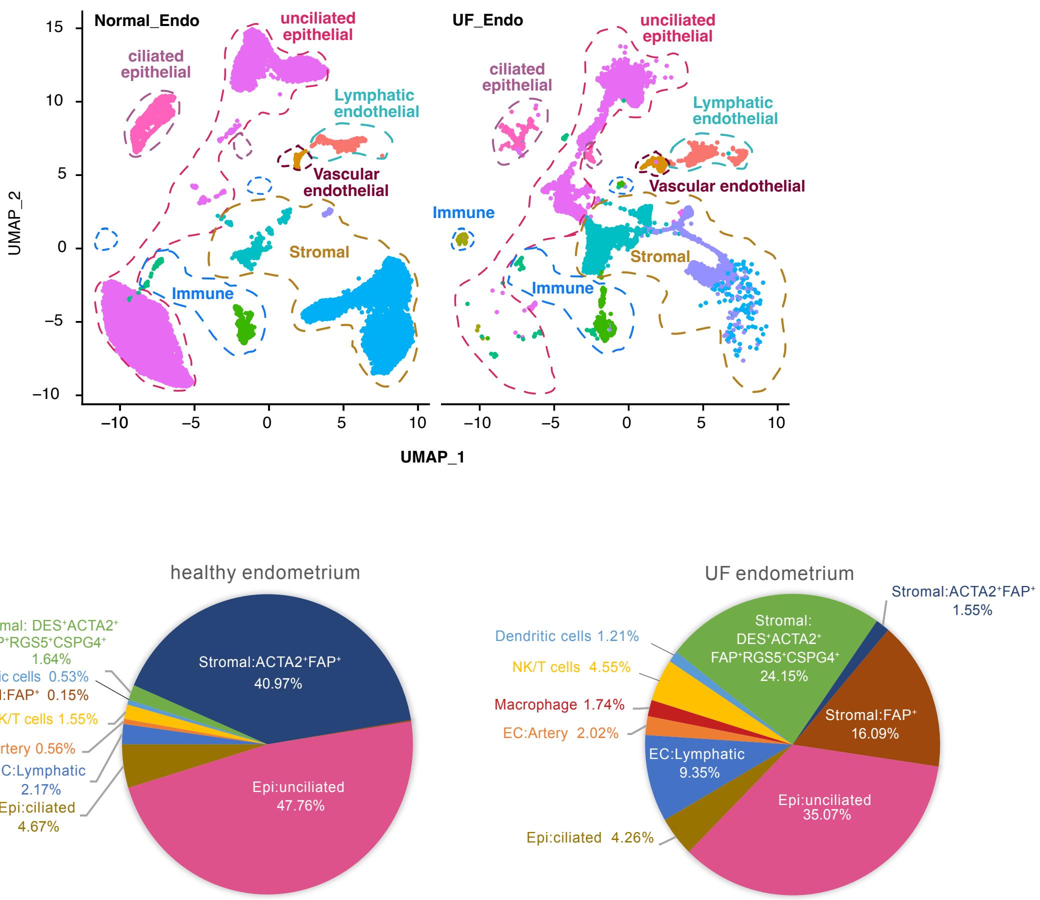


**Supplementary Fig. 10 Differences of cell type composition between healthy and UF endometrium datasets at single cell level. a** UMAPs of healthy (left) and UF HMB (right) endometrium, highlighting differences between disease and healthy endometrium tissue. **b** Percentage of annotated cell clusters in healthy (left) and UF (right) endometrium.

**Supplementary Fig. 11: Collagen and laminin signalling in healthy and UF endometrium at single cell level.**

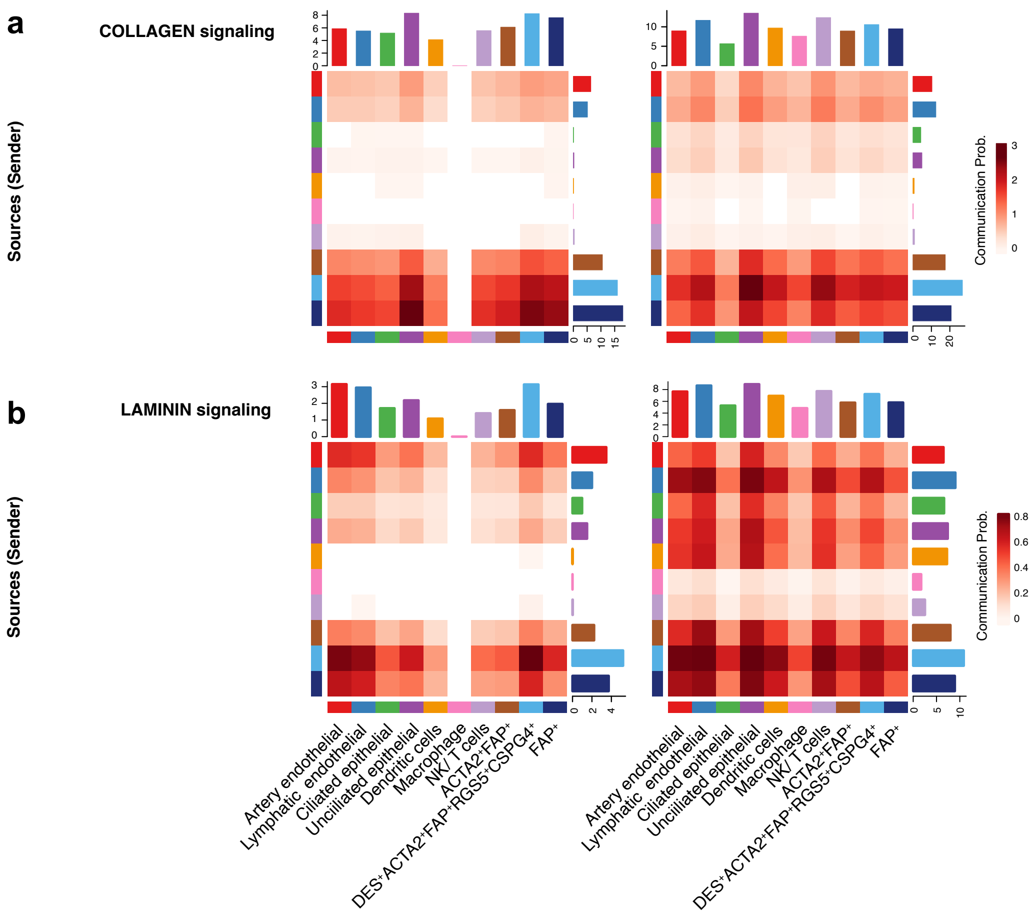


**Supplementary Fig. 11 Collagen and laminin signalling in healthy and UF endometrium at single cell level.**
Heatmap of **a** collagen and **b** laminin signalling between single cell clusters in control (left) and UF (right) endometrium are shown. The strength of interactions of ligand-expressing clusters (y-axis) and receptor-expressing clusters (x-axis) are presented in the heatmap. The bar plots represent the overall expression level of ligands (at the left side of the heatmap) or receptors (at the top of the heatmap) in the corresponding clusters.

## Supplementary Fig. 12: Comparison of signalling pathways in healthy and UF tissues.

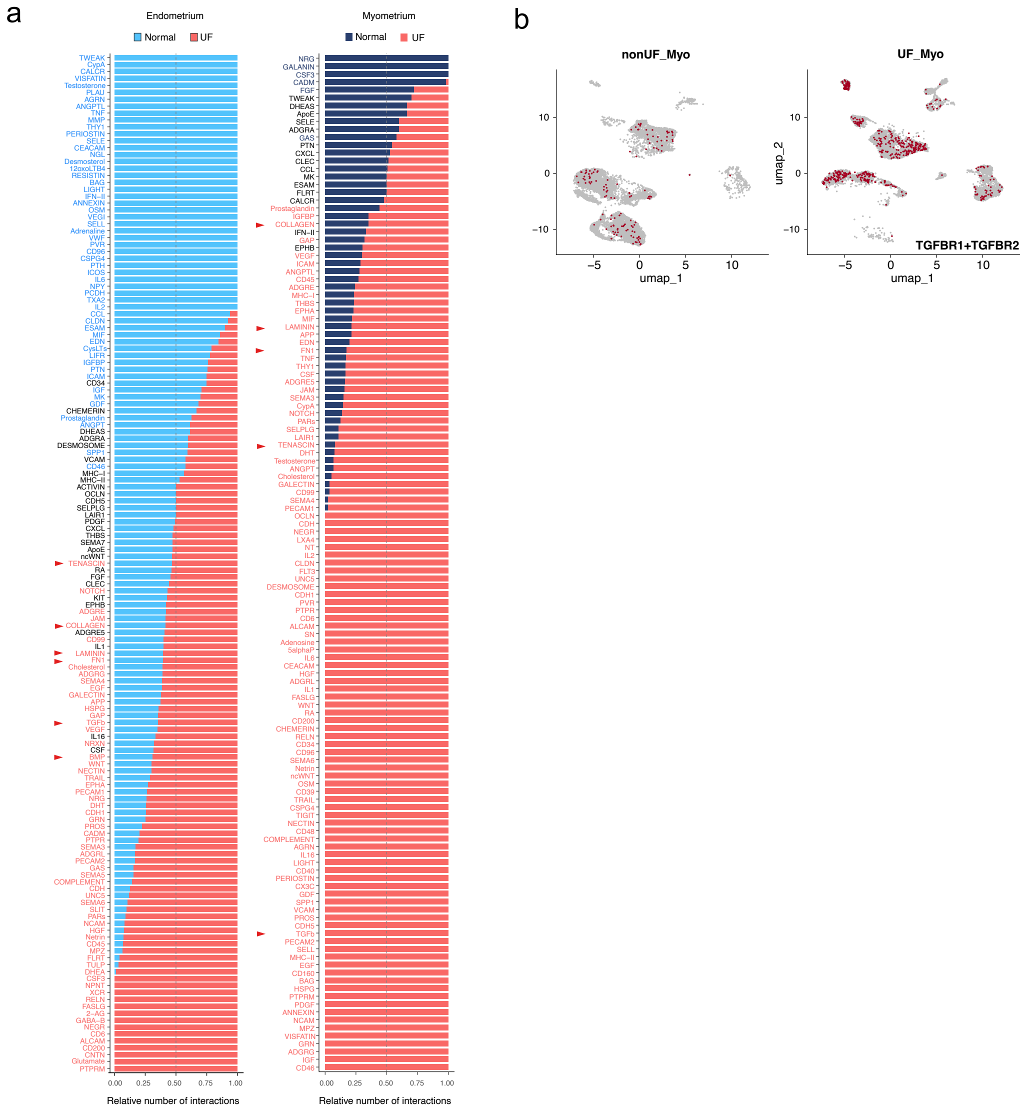


**Supplementary Fig. 12  Comparison of signalling pathways in healthy and UF tissues.** **a** Stacked barplot showing relative ratio of interactions in each signalling pathway predicted in the endometrium (left) and myometrium (right) obtained from normal (blue) and UF (red) donors. Triangles in red highlight the signalling pathways known to be elevated in leiomyoma. **b** Feature plot showing the expression of *TGFBR1* and *TGFBR2* in normal (left) and UF (right) myometrium.

**Supplementary Fig. 13: Enriched pathways of genes involved in THESC decidualization process identified by using bulk short read and long read sequencing.**

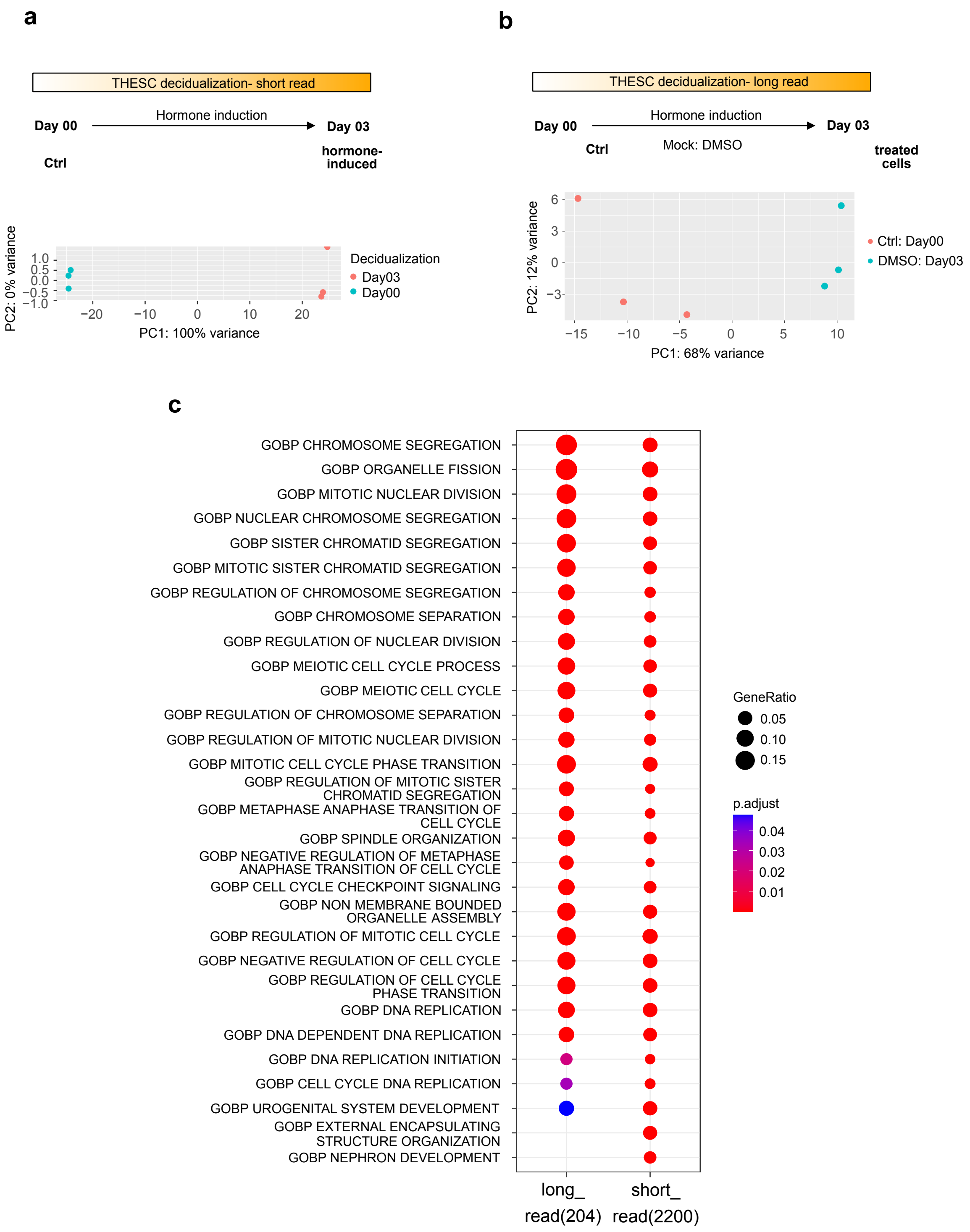


**Supplementary Fig. 13 Enriched pathways of genes involved in THESC decidualization process identified by using bulk short read and long read sequencing.** PCA plots of THESC decidualization in **a** Illumina short read RNA sequencing and **b** Nanopore long read sequencing. **c** Dot plot of the enriched pathways in the decidualization by GSEA analysis using MSigDB C5 GO:BP database.

**Supplementary Fig. 14: Enriched pathways of genes with alternative isoform usage identified in TGF-β treated THESC during decidualisation.**

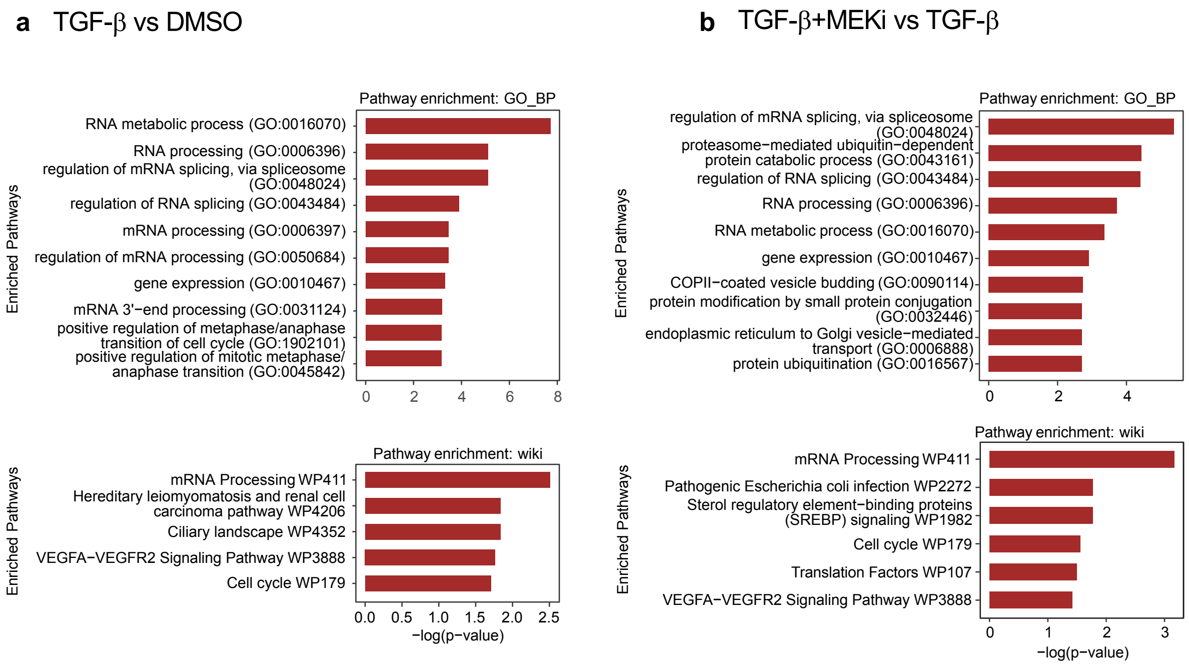


**Supplementary Fig. 14 Enriched pathways of genes with alternative isoform usage identified in TGF-β treated THESC during decidualisation.**
**a** Comparison between DMSO vs TGF-β treatment. **b** TGF-β vs MEKi + TGF-β using GO-BP database (upper panel) or wiki pathway database (bottom panel).
