# Supplementary material for: A systems-based approach to uterine fibroids identifies differential splicing associated with abnormal uterine bleeding
Source: Commun Med (Lond). 2025 Jul 31;5:318. doi: 10.1038/s43856-025-01051-x (PMC12311048; doi:10.1038/s43856-025-01051-x)
Supplement: Supplementary file 3 — Description of Additional Supplementary Files [file 43856_2025_1051_MOESM3_ESM.pdf]

Description of Additional Supplementary Files

File name- Supplementary Data 1

File description- Clinical information of 91 donors in this study

File name- Supplementary Data 2

File description- Identified variants with predicted impacts on protein function

File name- Supplementary Data 3

File description- Samples used in MOFA analysis

File name- Supplementary Data 4

File description- Transcriptomics and Proteomics data for MOFA analysis

File name- Supplementary Data 5

File description- Panel for targeted sequencing of fibroids

File name- Supplementary Data 6

File description- Source data for graphs in Figs 1-5 in the main manuscript
